# The Effect of Short-Term *Rhodiola rosea* Supplementation on Simulated Game Time, Perceived Fatigue, and Performance in Basketball Players

**DOI:** 10.3390/nu17233694

**Published:** 2025-11-25

**Authors:** Jing Wang, Haotian Zhao, Longqi Yu, Kai Zhao, Wei Jiang, Shuning Liu, Jin Dai, Lina Xu, Peng Sun, Hezhang Yun, Chang Liu

**Affiliations:** 1 School of Physical Education, Zhejiang Guangsha Vocational and Technical University of Construction, Jinhua 322100, China; wangjing@zjnu.edu.cn; 2 School of Sport Science, Beijing Sport University, Beijing 100084, China; 2019210252@bsu.edu.cn; 3 Department of Physical Education, Jiangnan University, Wuxi 214122, China; 4 The School of Arts, Beijing Sport University, Beijing 100084, China; 5 China Volleyball Academy, Beijing Sport University, Beijing 100084, China; 6 Sport Coaching College, Beijing Sport University, Beijing 100084, China; 7 Physical Education Department, Dalian Medical University, Dalian 116044, China; 8 Faculty of Health Sciences and Sports, Macao Polytechnic University, Macao 999078, China

**Keywords:** aerobic exercise, antioxidant capacity, basketball players, ergogenic aid, maximal oxygen uptake, perceived fatigue, *Rhodiola rosea*, simulated game, sports performance, VO_2_max

## Abstract

**Objectives**: This study aims to evaluate the effects of short-term *Rhodiola rosea* supplementation on simulated game performance, perceived fatigue, and aerobic capacity in basketball players. **Methods**: A total of 48 professional male basketball players participated in this study. The participants were randomly divided into a *Rhodiola rosea* group (RHO group, *n* = 24) and a Control group (CTR group, *n* = 24). During the experiment, the RHO group received continuous 28-day *Rhodiola rosea* supplementation, while the CTR group received empty capsules without being informed. Statistical analysis was performed using SPSS 22.0 software. A two-way mixed ANOVA (2 × 2) group (CTR/RHO) × time (Pre-test/Post-test) was conducted to compare differences. **Results**: In comparison to the CTR group, the RHO group showed significant differences in total completion time in the simulated game (*p* = 0.046), heart rate 60 s after the simulated game (*p* = 0.026), 5 km run (*p* = 0.027), YO-YO test (*p* = 0.036), VO_2_max (*p* = 0.034) and total antioxidant capacity (*p* = 0.044) compared to the CTR group. Within the RHO group, pre- and post-test comparisons revealed significant improvements in total completion time (*p* = 0.000), heart rate 30 s (*p* = 0.021) and 60 s (*p* = 0.016) after the simulated game, RPE score (*p* = 0.030), Countermovement jump test (*p* = 0.036), 5 km run (*p* = 0.000), YO-YO test (*p* = 0.000), VO_2_max (*p* = 0.000) and Superoxide Dismutase (*p* = 0.011). **Conclusions**: Short-term *Rhodiola rosea* supplementation can improve sprint performance and reduce perceived fatigue in basketball players during simulated games, enhance aerobic work capacity, and delay fatigue.

## 1. Introduction

Basketball is a sport that highly depends on teamwork. In a basketball game, having good physical fitness is crucial for athletes to achieve optimal performance and prevent sports injuries [1]. With the continuous development of skills and tactics, the speed and pace of modern basketball offense have accelerated, defensive areas have expanded, and man-to-man defense has become more rigorous, resulting in games characterized by high speed, high intensity, and high confrontation [2,3]. These developments demand that players possess superior aerobic and anaerobic capacities, the ability to perform repeated sprints, rapid changes of direction, and efficient recovery during short rest intervals between high-intensity bouts. However, such physical demands often lead to fatigue accumulation, and a subsequent decline in athletic performance, thereby placing greater strain on athletes’ physical conditioning.

Fatigue not only affects athletes’ immediate performance but also increases the risk of sports injury [4,5]. In modern elite basketball competitions, athletes are frequently required to compete consecutive games with limited recovery time [2]. Previous studies have shown [6] that back-to-back games can lead to significantly higher perceived fatigue and reduced overall performance in the second match. Moreover, analyses of NBA data have revealed [7] that higher game loads and fatigue levels are associated with an increased risk of injuries, with back-to-back schedules being particularly detrimental. Therefore, maintaining optimal physical fitness and ensuring efficient recovery are crucial for basketball players to achieve peak performance and reduce the likelihood of injury.

In high-intensity competitive sports, the use of nutritional supplements has become an effective strategy to enhance athletic performance and delay exercise-induced fatigue [8,9]. *Rhodiola rosea* (RHO) is a classic traditional herbal plant that grows in the rocky crevices and coastal cliffs of North America and the Asia and Arctic regions of Europe, and it has attracted attention for its effective anti-fatigue and performance-enhancing effects [10,11].Modern medical research has found that RHO is a nutritional supplement with anti-hypoxia and anti-fatigue properties that can significantly improve athletes’ physical and mental states [12,13,14,15]. De Bock et al. [16] conducted a 4-week RHO intake study on 24 young healthy participants and found that RHO could significantly enhanced time to exhaustion, VO_2_peak and VCO_2_peak, and thereby improving overall endurance exercise performance among the participants. Skarpanska-Stejnborn et al. [17] supplemented 11 professional rowing athletes with RHO and found that it increased the antioxidant levels in the plasma of these athletes. Additionally, animal experiments have shown [14] that RHO administration enhances swimming endurance in mice, elevates hepatic superoxide dismutase levels, and increases serum lactate dehydrogenase activity, concurrently decreasing serum urea nitrogen concentrations.

These findings collectively suggest that RHO, as a dietary supplement, can enhance athletic performance. However, it is important to note that most existing studies have focused primarily on endurance sports and laboratory-based tests, such as cycling or treadmill protocols, which may not accurately reflect the physiological demands of fast-paced, intermittent sports like basketball. Basketball is characterized by its intermittent, multi-directional, and high-intensity nature, making it challenging to directly assess performance and fatigue under real competitive conditions. Consequently, researchers often employ controlled “simulated game” protocols to replicate the intensity and demands of actual competition for performance assessment [18]. Nonetheless, research investigating the effects of RHO supplementation on the athletic performance and post-fatigue recovery of basketball players remains scarce to date.

In summary, although existing evidence supports *Rhodiola rosea* as a promising ergogenic and anti-fatigue supplement in endurance sports, research on its acute effects during simulated competition and its impact on perceived fatigue among basketball players remains limited. Therefore, this study aimed to examine the effects of short-term RHO supplementation on simulated game performance, perceived fatigue, and physical performance in basketball players, providing a theoretical basis for its potential role in enhancing basketball performance and delaying fatigue perception.

## 2. Materials and Methods

### 2.1. Participants

The research included 48 professional male basketball players from Beijing Sport University. The experiment began in June 2024 and was conducted at Beijing Sport University. Participants were randomly allocated into two groups: the RHO group (*n* = 24) and the CTR group (*n* = 24). All included participants had a certain level of sports experience and training, and all were national level 2 athletes or above. Participant profiles are summarized in Table 1. Participants agreed not to take RHO, additional nutritional supplements, or alcohol-containing products until all experiments were completed, to ensure no unnecessary interference during the experiment. In addition, all participants were abstained from tobacco use and were not undergoing antidepressant therapy or stimulant drugs during the study, and were healthy with no chronic diseases, no neuromuscular or musculoskeletal diseases related to sports, cardiovascular diseases, or diabetes. Written informed consent was obtained from all participants, confirming their comprehension of the study and voluntary participation. This study was approved by the Sports Science Experiment Ethics Committee of Beijing Sport University (No. 2024219H), and the approval date was 21 June 2024.

### 2.2. Experimental Procedure

**Figure 1:**
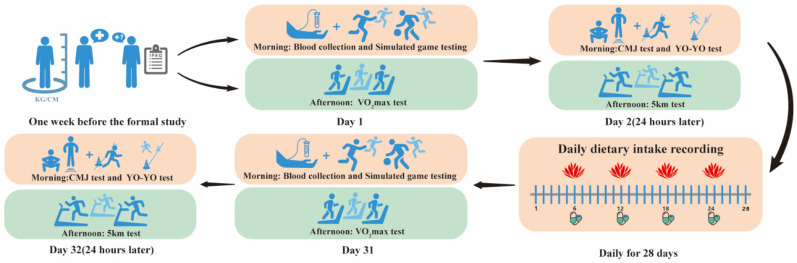
Diagram of the Experimental Procedure.

This study adopted a randomized, controlled, double-blind study, and participants were instructed to fast for at least 8 h the night before the start of the study. All participants were called to the site for a health visit one week before the study began. Each participant completed a questionnaire to assess their physical activity intensity, providing an in-depth understanding of their physical activity level. Height and weight were also measured. The methodological workflow is illustrated in Figure 1. After the tests, RHO was distributed to the athletes, who were instructed to take it continuously for 28 days. Daily text messages were sent to remind the athletes to take their supplements on time. Dietary intake of the participants was recorded during this period. RHO was obtained from Beijing Tong Ren Tang Pharmaceutical Co., Ltd. (Beijing, China), containing 0.5 g of salidroside per 100 g of raw material. Participants in the RHO group received a total daily dose of 2.4 g, administered in a fasted state 30 min before breakfast and lunch. For the CTR group, RHO powder was removed from the capsules, which were ingested under fasting conditions without participants’ knowledge. To minimize placebo effects, all participants consumed an equal volume of water with each administration.

### 2.3. Daily Dietary Intake Recording

In order to minimize potential confounding effects arising from variations in dietary patterns on the current investigation, we recorded the diet of all participants throughout the study. We provided multiple meal options for the participants to choose from, all of which met the relevant national food safety standards. During the study dietary intake (kcal), carbohydrates, proteins, and fats were recorded.

### 2.4. Blood Collection

The participants underwent blood collection twice, both before the simulated game. Before each collection, participants fasted for 8–12 h and consumed a light meal the evening before. Venous blood samples were uniformly collected between 8:00 and 8:30 a.m. During the procedure, participants were seated with their arms placed on a stable platform, ensuring full exposure of the venipuncture site. Following collection, tubes were gently inverted five times to mix, then placed on ice. After completing blood collection for all participants, samples were centrifuged at 1000× *g* for 10 min at 4 °C to separate plasma. The resulting red blood cells were washed three times with cold isotonic saline (4 °C). Plasma and red blood cells were stored at −80 °C until biochemical analyses were performed.

Plasma total antioxidant capacity (TAC) was determined (Randox-TAS, NX 2332, Randox, Crumlin, UK). Superoxide dismutase (SOD) activity was assessed (Randox-SOD, SD125, Randox, Crumlin, UK). Plasma malondialdehyde (MDA) concentration, an indicator of lipid peroxidation, was measured with the thiobarbituric acid reactive substances assay, with absorbance measured at λ = 532 nm using a multi-mode microplate reader. Plasma creatine kinase (CK) activity was analyzed (Alpha Diagnostic Int., San Antonio, TX, USA) by spectrophotometry.

### 2.5. Simulated Game Testing

**Figure 2:**
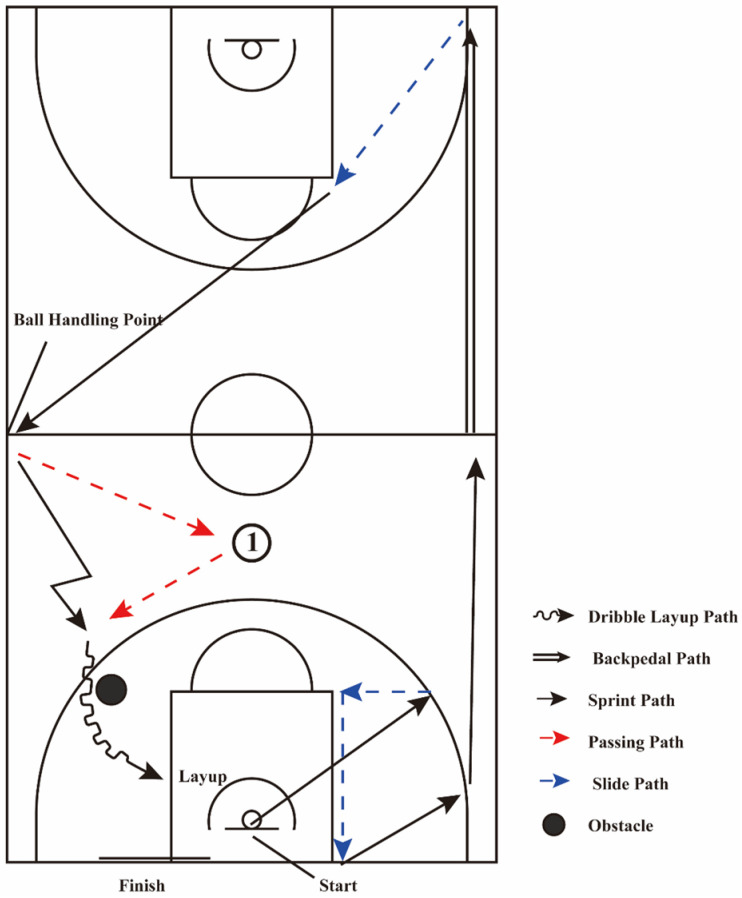
Diagram of the Simulated Game.

Before starting the test, participants attended a training session where they practiced each part of the test and learned the movements and rules required for each segment. All tests were conducted at the same time and under the same conditions. Before each test, players performed their usual warm-up routines. The Simulated Game procedure is shown in Figure 2.

Participants quietly waited on the test site for 3–5 min until their HRs stabilized before starting the test. The simulated game test consisted of three parts:

Part 1: Participants performed a standing vertical jump at the center of the hoop (only during the first run of the test). After landing, two instructors started timing. Participants completed sprints (indicated by a black solid single arrow in Figure 2), defensive slides (indicated by a blue dashed arrow in Figure 2), and backpedaling (indicated by two black solid arrows in Figure 2) at maximal speed. This part included 4 sprints (sprint 1, sprint 2, sprint 3, sprint 4), 3 defensive slides (slide 1, slide 2, slide 3), and 1 backpedal.
Part 2: An assistant stands at the left sideline midcourt with the ball. Upon receiving it, the participant passes the ball to an assistant at the top of the three-point arc (as depicted in Figure 2, Position ①, where the red dashed line represents the basketball passing path), then quickly moves to receive the return pass and completes a dribble layup, with the player’s movement path is indicated by a polyline, and the dribble-layup path is shown as a wavy line.
Part 3: After the layup, participants moved to the baseline for a 5 s rest. They then started from directly under the hoop. These three parts constituted one set, with a total of three sets. During the test, instructors recorded the time for each activity path. After the simulated game, they recorded the ratings of perceived exertion (RPE).

### 2.6. RPE Assessment

Basketball players rated their RPE [19] immediately following the simulated game, and the scores were recorded. The entire simulated game process was videotaped from the stands using a Sony (Sony Corporation, Minato, Japan) FDR-AX700 camera.

### 2.7. HR Test

Before the simulated game, a HR monitor (Polar, Kempele, Finland) was employed to record the athletes’ HR changes. The HR monitors were distributed before the start of the simulated game, and the study staff assisted the athletes in wearing the monitors. After putting on the HR monitor, the participants remained quiet for 3–5 min until their HRs stabilized before starting the simulated game test. The HRs at 30 s and 60 s after the simulated game were also recorded to understand fatigue recovery.

### 2.8. YO-YO Test

**Figure 3:**
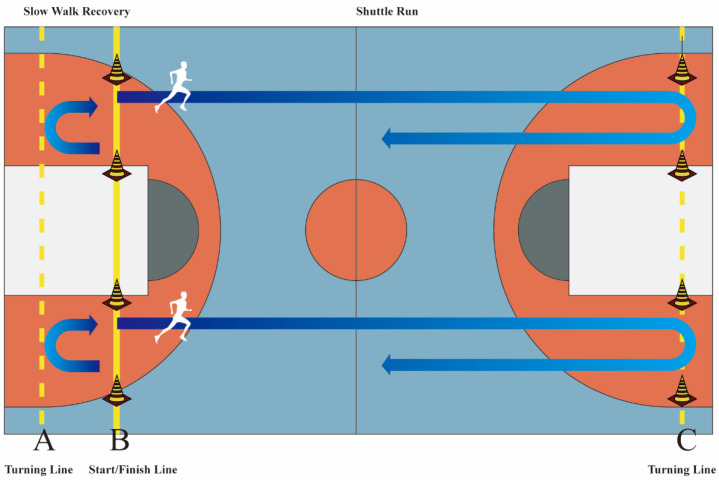
Diagram of the YO-YO Testing.

The yo-yo test used in this experiment was the Yo-Yo Intermittent Recovery Test, Level 1. Before the test, three lines were marked on the basketball court: two turning lines labeled A and C, and one start/finish line labeled B. The distance between the turning lines and the start/finish line, B–C, was 20 m. Participants were required to perform shuttle runs on the 20-m straight track. After completing 40 m, there was a 5-m slow walk recovery area (A–B). Each completion was followed by a 10-s recovery period during which participants had to walk or jog from line B to line A and back to line B. The speed and prompts during the test were controlled by the YYIR1 test recording. The specific content of the test was shown in Figure 3. At both ends of the turning lines, monitors were arranged to manage the test. The first time a participant failed to complete the shuttle run within the specified time, they received a warning. If a participant failed to complete the shuttle run within the specified time for two consecutive attempts, the test was considered over, and the total distance was recorded.

### 2.9. CMJ Test

Participants performed the CMJ starting from a standing position with feet shoulder-width apart, hands at their sides, and torso upright on the center of the force plate. Upon the tester’s command “jump”, they rapidly squatted by bending the hips and knees and immediately jumped vertically with maximal effort. Hand position remained unchanged, the upper body stayed upright, and the hips, knees, and ankles were fully extended throughout the movement. Three valid jumps were performed with a one-minute recovery between attempts. CMJ height was recorded in centimeters.

### 2.10. VO_2_max Test

**Figure 4:**
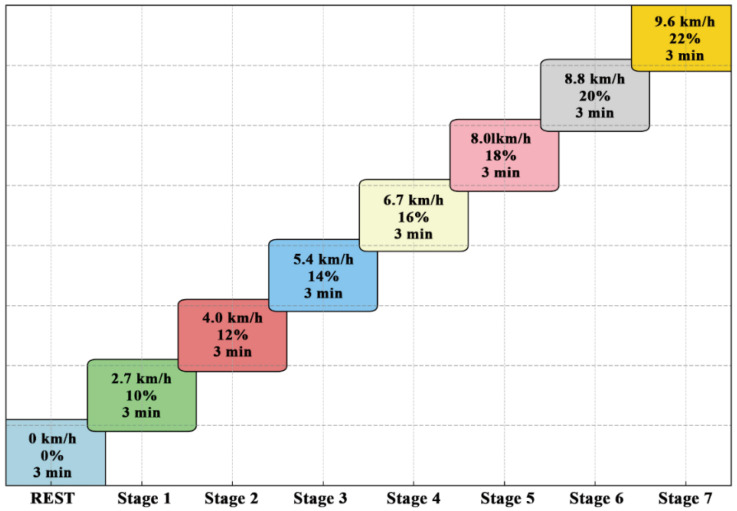
Bruce Treadmill Load Scheme.

Before testing, the equipment was preheated and gas calibration was performed to ensure proper functioning. Participants completed a standardized warm-up, were fitted with Polar HR monitors and rested for 3 min to record baseline data. The exercise test followed the Bruce treadmill protocol, with speed and incline increased every 3 min until volitional exhaustion (specific test details are shown in Figure 4). Throughout the test, a tester continuously monitored the participants, provided verbal guidance to maintain a steady pace, and offered encouragement to maximize effort.

Upon volitional exhaustion, HR, perceived fatigue, and exercise duration were recorded. Exercise termination criteria included: RPE ≥ 18, respiratory quotient ≥ 1.10, ≥90% of HRmax; or an oxygen uptake plateau (<2 mL·kg^−1^·min^−1^ change over 2 min). Meeting three of these criteria indicated VO_2_max. The test was immediately stopped if participants exhibited dyspnea, distress, shaking, or pallor to ensure safety.

### 2.11. 5 km Test

Participants were instructed to complete a 5 km run as quickly as possible on a treadmill set at 1% incline, controlling their own pace. To prevent pacing strategies, visual access to treadmill controls (incline) and display information (speed, elapsed time) was blocked using custom screens.

### 2.12. Data Analysis

The research data were entered in Excel and analyzed using SPSS software (version 26, IBM Corp., Armonk, NY, USA), with data visualization performed in GraphPad Prism (version 9.0, GraphPad Software, Boston, MA, USA). Continuous variables are presented as mean ± SD. Normality was assessed using the Shapiro-Wilk test. A 2 × 2 mixed-design ANOVA [group (CTR vs. RHO) × time (Pre-test vs. Post-test)] was conducted to evaluate intervention effects. SportScode software (Hudl, Lincoln, NE, USA) was used to analyze the completion time of each route in the simulated competition frame by frame.

## 3. Results

### 3.1. Daily Recorded Intake of Carbohydrates, Proteins, Fats, and Energy

**Figure 5:**
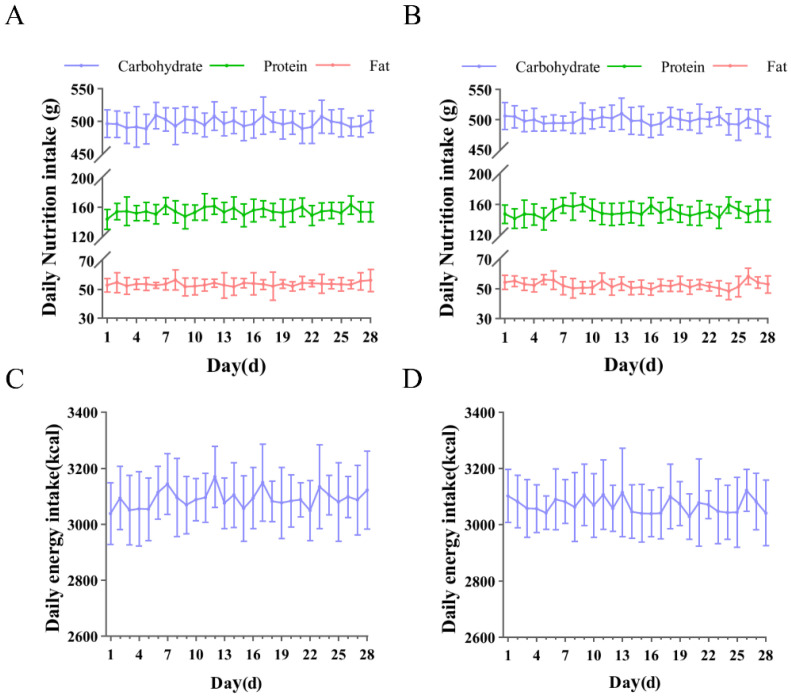
Daily recorded intake of carbohydrates, proteins, fats, and energy. Daily nutrient intake (**A**) and energy intake (**C**) of the RHO group; daily nutrient intake (**B**) and energy intake (**D**) of the CTR group, g = grams, kcal = kilocalories.

To avoid the potential impact of dietary factors on the experimental results, we provided each participant with several fixed meal options to choose from. The dietary intake of the participants is shown in Figure 5. The dietary intake of participants in both the RHO group and the CTR group was approximately 498 g of carbohydrates, 152 g of protein, and 53 g of fat, resulting in a total energy intake of around 3085 kilocalories.

### 3.2. Blood Analysis Results

**Figure 6:**
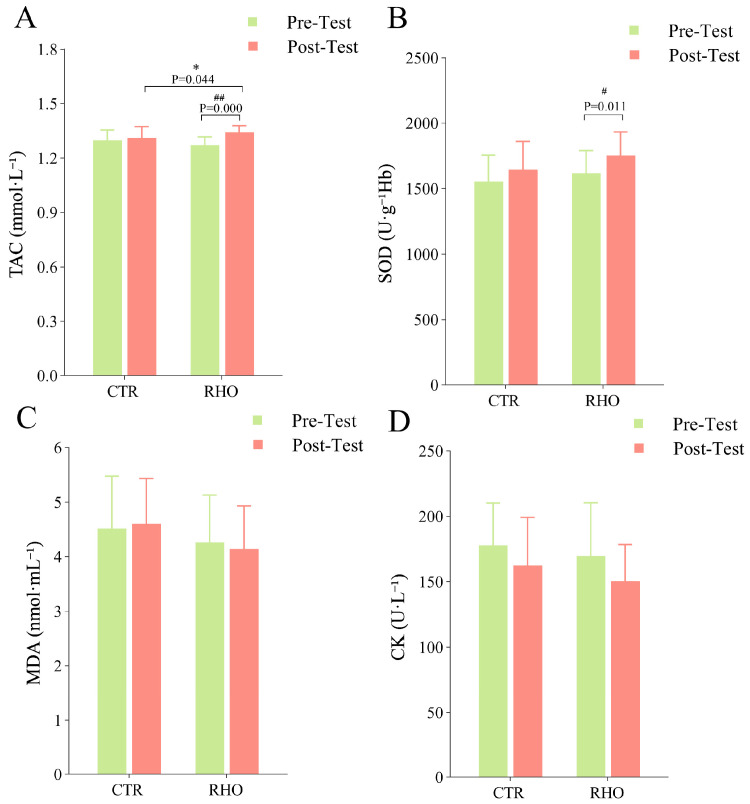
Blood Analysis Results. (**A**): Total Antioxidant Capacity (TAC); (**B**): Superoxide Dismutase (SOD); (**C**): Malondialdehyde (MDA); (**D**): Creatine Kinase (CK); * *p* < 0.05, between-group difference; # *p* < 0.05, ## *p* < 0.01, within-group difference between pre- and post-test, CTR = Control group, RHO = Rhodiola group.

Hematological parameters of the athletes are illustrated in Figure 6. The RHO group demonstrated a statistically significant increase compared to the CTR group in TAC values (*p* = 0.044, 95% CI = [−0.060, −0.001], ηp^2^ = 0.001). Relative to pre-test, the RHO group exhibited significant increases in both TAC and SOD following supplementation (*p* = 0.000, 95% CI = [−0.102, −0.040], ηp^2^ = 0.241), (*p* = 0.011, 95% CI = [−235.346, −32.179], ηp^2^ = 0.175). No significant differences were observed in MDA or CK levels either within or between groups after 28 days of continuous RHO supplementation.

### 3.3. Results of the Simulated Game Tests for Athletes

#### 3.3.1. Completion Time of Each Stage of the Simulated Game

**Figure 7:**
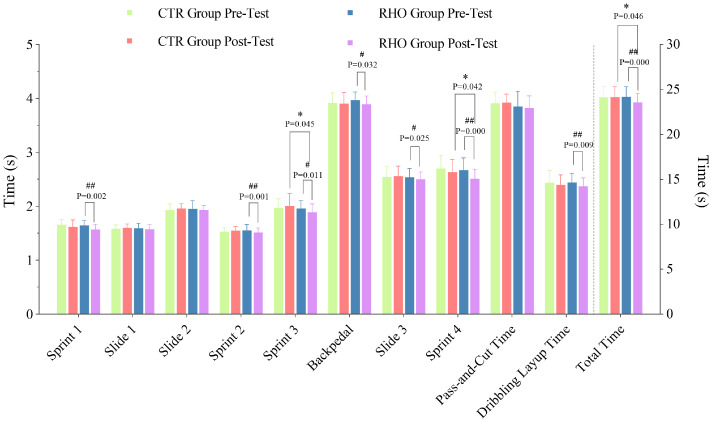
Time variations for each stage of the simulated game. * *p* < 0.05, between-group difference; # *p* < 0.05, ## *p* < 0.01, within-group difference between pre- and post-test, s = seconds, CTR = Control group, RHO = Rhodiola group.

The completion times for each stage of the simulated game are presented in Figure 7. Overall, RHO group demonstrated superior performance during the sprint stage. Statistical analysis revealed a significant disparity between the RHO and CTR groups during sprint 3 (*p* = 0.045, 95% CI = [0.002, 0.230], ηp^2^ = 0.035), sprint 4 (*p* = 0.042, 95% CI = [0.005, 0.244], ηp^2^ = 0.033) and total completion time (*p* = 0.046, 95% CI = [0.010, 1.184], ηp^2^ = 0.019).

Within the RHO group, significant improvements from pre- to post-test were ob-served in sprint 3 (*p* = 0.011, 95% CI [0.017, 1.122], ηp^2^ = 0.015), backpedaling (*p* = 0.032, 95% CI [0.007, 0.147], ηp^2^ = 0.059) and slide 3 (*p* = 0.025, 95% CI [0.005, 0.069], ηp^2^ = 0.020). Highly significant improvements were found in sprint 1 (*p* = 0.002, 95% CI [0.032, 0.132], ηp^2^ = 0.195), sprint 2 (*p* = 0.001, 95% CI [0.018, 0.064], ηp^2^ = 0.029), sprint 4 (*p* = 0.000, 95% CI [0.097, 0.225], ηp^2^ = 0.353), dribbling layup time (*p* = 0.009, 95% CI [0.019, 0.124], ηp^2^ = 0.152) and total completion time (*p* = 0.000, 95% CI [0.446, 0.785], ηp^2^ = 0.366).

#### 3.3.2. Changes in Athletes’ HR During Simulated Game

**Figure 8:**
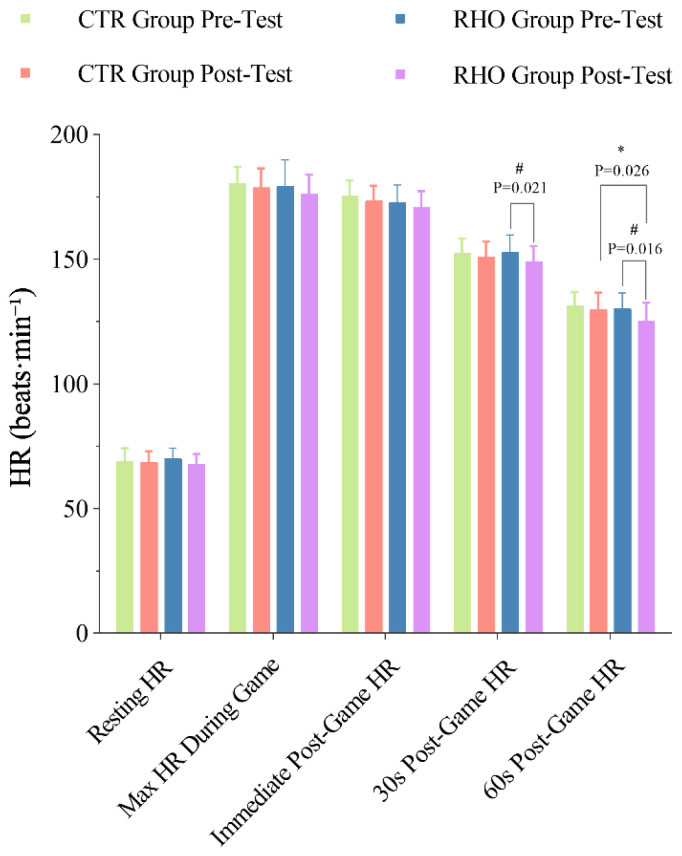
Changes in HR during the simulated game. * *p* < 0.05, between-group difference; # *p* < 0.05, within-group difference between pre- and post-test, HR = heart rate, CTR = Control group, RHO = Rhodiola group.

The results of resting HR before the simulated game, maximal HR during the game, and HR immediately, 30 s, and at 60 s after the game are presented in Figure 8. Compared with the pre-test, the RHO group exhibited significantly lower HR at 30 s (*p* = 0.021, 95% CI [0.624, 7.210], ηp^2^ = 0.019) and 60 s (*p* = 0.016, 95% CI = [1.001, 9.082], ηp^2^ = 0.106) after the simulated game. The RHO group exhibited a markedly reduced HR at the 60-s post-game when compared to the CTR group (*p* = 0.026, 95% CI = [0.577, 8.756], ηp^2^ = 0.019).

#### 3.3.3. Changes in Athletes’ Perceived Fatigue After Simulated Game

**Figure 9:**
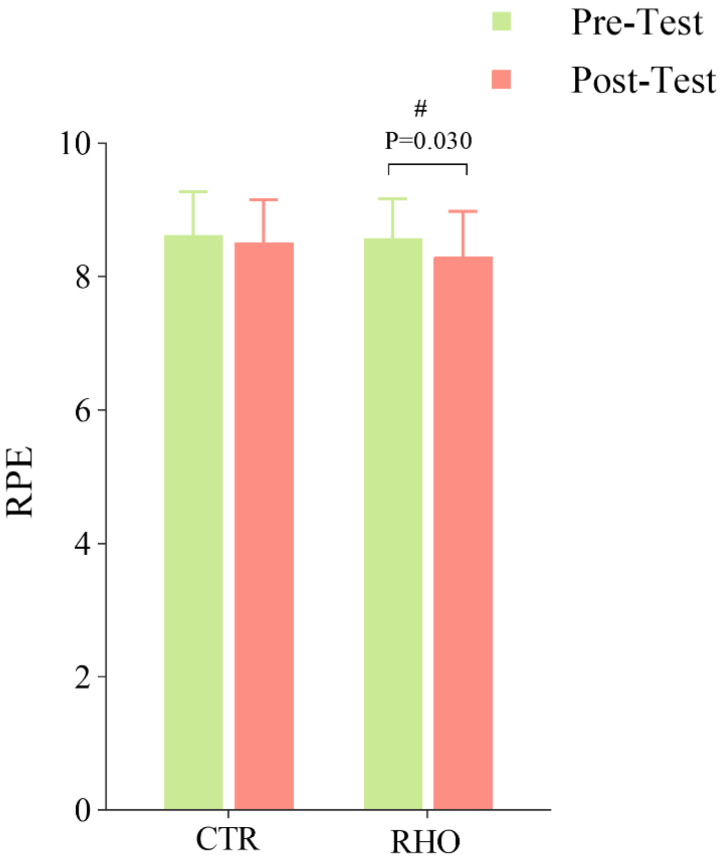
RPE scores of athletes after completing the simulated game. # *p* < 0.05, within-group difference between pre- and post-test, RPE = ratings of perceived exertion, CTR = Control group, RHO = Rhodiola group.

The RPE scores after the athletes completed the simulated game are shown in Figure 9. While no significant differences were detected between the RHO and CTR groups, the RHO group demonstrated a significant decrease in perceived fatigue levels relative to baseline measurements (*p* = 0.131, 95% CI = [0.025, 0.475], ηp^2^ = 0.019).

### 3.4. Test Results of Basketball Players’ Athletic Performance

#### 3.4.1. CMJ Test Results

**Figure 10:**
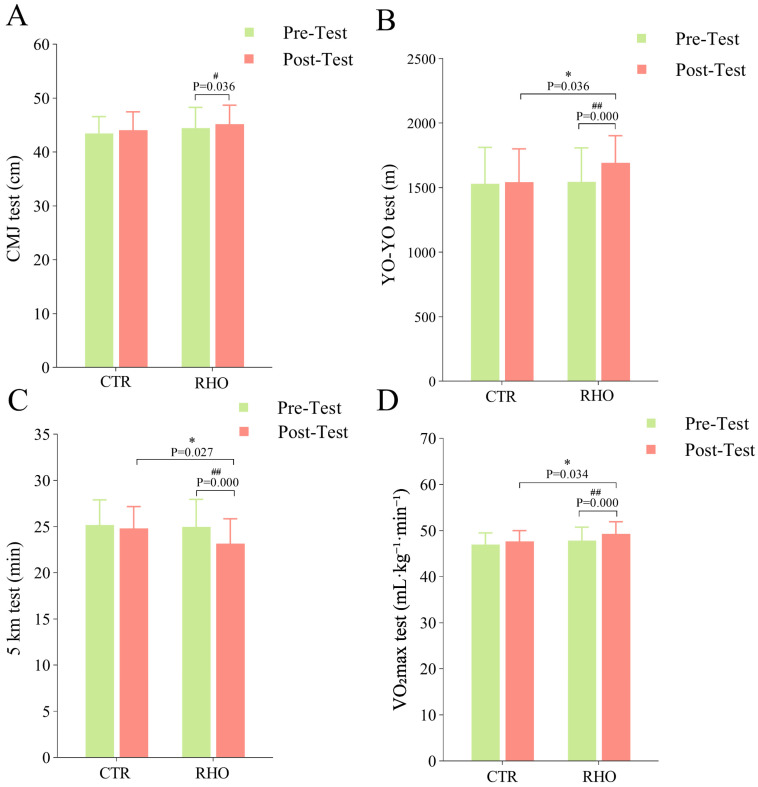
Measurement results of basketball players’ athletic performance. (**A**): CMJ test; (**B**): YO-YO test; (**C**): 5 km test; (**D**): VO_2_max test; * *p* < 0.05, between-group difference; # *p* < 0.05, ## *p* < 0.01, within-group difference between pre- and post-test, CTR = Control group, RHO = Rhodiola group.

The CMJ test results are shown in Figure 10A: The RHO group exhibited a significant improvement in CMJ height compared with the pre-test (*p* = 0.036, 95% CI = [−1.353, −0.047], ηp^2^ = 0.155), whereas no significant difference was found between the RHO and CTR groups.

#### 3.4.2. YO-YO Test Results

The final completion distance of the athletes in the YO-YO test is shown in Figure 10B. The RHO group demonstrated a significant improvement in completion distance compared with the pre-test (*p* = 0.000, 95% CI = [−180.220, −109.780], ηp^2^ = 0.476). A statistically significant distinction was noted when comparing the RHO and CTR group (*p* = 0.036, 95% CI = [−283.674, −9.659, −0.047], ηp^2^ = 0.027). The results indicate that continuous 28-day supplementation with RHO can improve the YO-YO test distance for athletes.

#### 3.4.3. 5 km Test Results

As shown in Figure 10C, the RHO group demonstrated a significant improvement in 5 km running time relative to the CTR group (*p* = 0.027, 95% CI = [0.195, 3.158], ηp^2^ = 0.034). Within the RHO group, completion time was also markedly reduced compared with the pre-test (*p* = 0.000, 95% CI = [1.171, 2.466], ηp^2^ = 0.335).

#### 3.4.4. VO_2_max Test Results

Aerobic capacity was evaluated using VO_2_max (Figure 10D). The RHO group exhibited a significantly higher VO_2_max relative to the CTR group (*p* = 0.034, 95% CI = [−3.089, −0.125], ηp^2^ = 0.060). Within the RHO group, VO_2_max also increased markedly from pre-test to post-test (*p* = 0.000, 95% CI = [−2.049, −0.676], ηp^2^ = 0.273), indicating a substantial improvement in aerobic capacity following supplementation.

## 4. Discussion

This study found that short-term supplementation with RHO significantly shortens the time it takes athletes to complete a simulated game and reduces their HR at 30 s and 60 s post-game. It also accelerates HR recovery and improves perceived fatigue after the simulated game, while enhancing aerobic capacity. These findings provide theoretical support for the potential performance-enhancing effects of RHO in basketball team projects. Therefore, basketball players may consider including RHO as part of their ergogenic supplementation strategies to optimize performance and recovery.

To better simulate athletes’ anaerobic system energy supply performance under high-intensity competition, the intensity of the game was increased. At this intensity, ATP production mainly comes from phosphocreatine stores and glycolytic capacity. Therefore, the rate of ATP generation in the energy system is one of the key factors affecting the completion time of the simulated game. Ballmann, C.G. et al. [20] found that short-term RHO supplementation increased both average and peak power output in athletes, thereby enhancing anaerobic exercise capacity. The underlying mechanism has been suggested to be that RHO can activate ATP synthesis or resynthesis in mitochondria, enhance the oxidative phosphorylation process, inhibit glycolysis, and stimulation of post-exercise energy recovery, thereby delaying fatigue during high-intensity exercise [21,22]. However, conflicting evidence exists regarding the effects of RHO supplementation on phosphocreatine recovery during exercise-induced fatigue, with some studies demonstrating no significant impact [23]. These findings suggest that further investigation is warranted to elucidate the mechanisms by which RHO enhances anaerobic exercise performance.

The post-test 30-s and 60-s recovery HR of the RHO group athletes during the simulated game were lower than the pre-test HRs, and their RPE was significantly reduced, indicating reduced myocardial oxygen consumption and improved cardiac reserve. Spasov et al. found that 20 days of daily 100 mg RHO supplementation allowed participants to complete higher exercise loads at the same HR, and they also found that post-exercise HR recovery in the RHO group was significantly faster [24]. These results are similar to those of this study. This may be because RHO can inhibit monoamine oxidase [25], which degrades monoamine substances in the brain, such as dopamine and serotonin [26]. Increased dopamine levels can also increase the production of endogenous opioids. Additionally, RHO can increase the sensitivity of endogenous opioid receptors. Opioids have been shown to reduce the effects of beta-adrenergic stimulation on myocardial cells and lower HR. This has also been confirmed in a time-dose relationship experiment with RHO supplementation and endogenous opioid receptor concentration [25]. This may also be the reason for the decreased perceived fatigue in athletes. Early experiments found that when participants took opioid receptor antagonists, their perceived fatigue during exercise increased significantly, and their performance decreased significantly [27,28]. Additionally, studies have shown that RHO supplementation can reduce post-exercise fatigue biomarkers, including lactate dehydrogenase, alanine aminotransferase, and aspartate aminotransferase [29], as well as creatine kinase [30]. This suggests that RHO has the effect of alleviating exercise-induced fatigue and muscle damage.

RHO supplementation can improve athletes’ performance in the CMJ test, YO-YO test, 5 km run, and VO_2_max test, especially in enhancing aerobic capacity. This is consistent with most current studies on the effects of RHO on aerobic capacity [14,16,31,32]. RHO exerts its effects primarily through modulation of the hypoxia-inducible factor 1 (HIF-1) signaling pathway. As a key transcription factor, HIF-1 regulates the expression of the erythropoietin (EPO) gene under hypoxic conditions, thereby promoting erythropoiesis and enhancing oxygen transport [31,33,34]. EPO is mainly produced by the kidneys and liver, traveling through the bloodstream to the erythroid progenitor cells in the bone marrow, where it binds to EPO receptors on their surface. This activates the downstream JAK2-STAT5 signaling pathway, regulating the expression of genes related to erythrocyte proliferation and differentiation [35,36]. This process increases the number of red blood cells in the blood, enhances oxygen transport capacity, and ultimately improves the body’s hypoxia tolerance and anti-fatigue ability. EPO has an intrinsic positive correlation with endurance performance in humans. Existing evidence indicates that four weeks of sustained RHO supplementation enhances endurance performance and increases peak oxygen consumption [34]. Moreover, RHO has been reported to reduce post-exercise fatigue, elevate cerebral dopamine concentrations, and serve as an ergogenic aid among elite athletes. It promotes skeletal muscle anabolism, enhances performance during exhaustive exercise, and facilitates cardiovascular recovery following exertion [11,17,21]. Despite these encouraging findings of the present study, several key physiological and molecular variables that could clarify the mechanisms of RHO action—such as HIF-1α, EPO, and opioid-related markers-were not assessed in this study. Therefore, the pathways discussed in the manuscript should therefore be interpreted as literature-based explanations rather than direct evidence from the present data of this study. Future studies incorporating biochemical and molecular measurements are warranted to verify these proposed mechanisms.

Several limitations of the present study should be acknowledged. First, the 28-day intervention may not reflect the effects of longer-term RHO supplementation, and thus the durability and time-course of the observed benefits remain uncertain. Although dietary intake was monitored and controlled during the trial, between-study differences in participants’ habitual nutrition and lifestyle behaviors may nevertheless have influenced the outcomes. Additionally, no correction for multiple comparisons was performed in this study. In subsequent large-sample confirmatory studies, correction for multiple comparisons will be adopted to further validate the robustness of the intervention effects.

## 5. Conclusions

Short-term supplementation with RHO can improve sprint performance and perceived fatigue in basketball players during simulated games, enhance aerobic capacity, and delay fatigue. Athletes may consider including this supplement in their daily regimen and taking an appropriate dosage during training to enhance performance.

## Figures and Tables

**Table 1:** Baseline characteristics.

Body Characteristics	CTR Group (*n* = 24)	RHO Group (*n* = 24)
Age (years)	20.33 ± 1.34	19.46 ± 2.02
Height (cm)	186.50 ± 6.11	188.29 ± 7.09
Weight (kg)	88.22 ± 6.05	91.33 ± 5.74
Body mass index (kg/m^2^)	25.37 ± 1.48	25.76 ± 1.90

## Data Availability

The datasets generated during and/or analyzed during the current study are available from the corresponding author on reasonable request, due to privacy considerations.
